# Perceived technology use, attitudes, and barriers among primary care nurses

**DOI:** 10.4102/hsag.v27i0.2056

**Published:** 2022-10-20

**Authors:** Million Bimerew, Jennifer Chipps

**Affiliations:** 1 School of Nursing, Faculty of Community and Health Sciences, University of the Western Cape, Cape Town, South Africa

**Keywords:** TAM, attitudes, barriers, nurses, health information technology, primary healthcare

## Abstract

**Background:**

In primary healthcare, health information technology has the potential to facilitate the delivery of healthcare services by improving quality of care, efficiency and patient safety. However, little is known about the uptake and technology acceptance among primary healthcare nurses.

**Aim:**

The aim of this study was to describe health information technology acceptance and use among primary healthcare nurses.

**Setting:**

Primary healthcare centres in the Western Cape.

**Methods:**

A quantitative descriptive survey was conducted with a sample of 160 nurses working in primary healthcare for more than 6 months, using a self-administered questionnaire based on the technology acceptance model constructs. Eighteen primary healthcare centres were randomly selected with a sample of 160 using nonprobability purposive sampling.

**Results:**

Ninety-three (58.1%) respondents completed the survey. Three-quarters of the respondents reported positive attitudes, positive perceptions of usefulness and ease of use towards the use of health information technology. Barriers of access and training were reported by 75%, with around half the respondents reporting poor computer and information accessing skills. Health information technology use was varied, with high ratings for seeking and using and low ratings of ability to use health information technology for patient administration and management. Health information technology use was predicted by perceptions of ease of use.

**Conclusion:**

This research presents a mixed picture of acceptance of technology among primary healthcare nurses and highlights the lack of access to computers and Internet in these settings.

**Contribution:**

This study contributes to the field of technology acceptance among primary healthcare nurses.

## Introduction

Health information technology has received much attention in the last decade, with the expansion of electronic health records, the expansion of digital technology and the availability of funding for implementation (Baillieu et al. 2020). Health information technology includes the use of any information and communication technology (ICT), m-health, using mobile phones for healthcare delivery or electronic health records (Rahimi et al. 2018) for patient outcomes such as enhancing patient safety using medication alerts, patient health outcomes tracking, recording medical history and diagnostic testing and making this information available for clinical decision support (Baillieu et al. 2020) and improving patient health (Carini et al. 2021).

The use of health information technology in primary healthcare is important for capturing health information at the first contact of care, thus contributing to continuity of care through improved communication among members of the healthcare team (Young & Nesbitt 2017). However, various barriers exist in the use of health information technology, specifically in low-income countries, such as a lack of infrastructure, cost, a lack of training or skilled human resources and system reliability (Akhlaq et al. 2016). Various studies have addressed factors influencing information-seeking behaviours of nurses towards the use of information technology in healthcare services (March, Vaikosen & Akporoghene 2020; Zigdon, Zigdon & Moran 2020), with studies finding that nurses working in resource-limited areas have little or no access to information technology to provide evidence-based patient care (Ahmad, Musallam & Allah 2018; Laki 2008). In addition, nurses expressed concerns such as the insufficient quantity of computers, content design, challenges with system capabilities and nurses’ computer knowledge and skills (Gaughan et al. 2022). This is further compounded by external factors such as work-related time pressure, computer literacy, technological competence (Vehko et al. 2019) and internal factors such as anxiety, fear and mixed attitudes towards technology (AlQudah, Al-Emran & Shaalan 2021; Ashtari & Bellamy 2021; Kuek & Hakkennes 2020; Metallo et al. 2022; Saleh et al. 2016).

A framework used to analyse technology acceptance is described in the technology acceptance model (TAM), which suggests a causal relationship between the constructs of attitudes, perceptions about usefulness (PU) and perceived ease of use of technology (PEU) and behavioural intention to use technology (Davis 1989; Rahimi et al. 2018). Thus, the use of health information technology is influenced by technology acceptance, which has been used to predict use of technology by different users (Gaughan et al. 2022) and different settings (Metallo et al. 2022). This model was identified as appropriate to describe the technology acceptance among primary healthcare nurses.

Considering the importance of primary healthcare and the benefits for continuity of care in this setting, the readiness, access to health information and technology acceptance in nurses in this setting is of vital importance. Although several studies have examined technology acceptance in nursing (Gagnon et al. 2012; Rho et al. 2015; Tubaishat 2018), only a few studies have specifically focused on primary healthcare (Gonçalves et al. 2016; Saleh et al. 2016; Watkins et al. 2018) and no studies of health information technology use and acceptance in nurses in primary healthcare in a low-resource setting such as South Africa were found. In the current health context in South Africa, with a quadruple burden of disease and a dedicated primary healthcare setting (Visagie & Schneider 2014), it is critical to identify the primary care nurses’ technology acceptance and use of health information technology in this setting.

### Aim of study

The aim of the study was to describe health information technology acceptance and use among primary health care nurses.

## Methods

A quantitative descriptive survey was conducted to collect data from a sample of 160 nurses working in 42 primary healthcare settings in the Western Cape. Eighteen primary healthcare centres were randomly selected for the study, and all categories of nurses who were employed in these clinical settings for more than 6 months at the time of the study were eligible for inclusion in the study (*n* = 160) using nonprobability purposive sampling. Exclusions for the study were nursing students and other health professions. All suitable respondents available on the day of data collection were approached for participation in the study. A self-administered paper-based questionnaire based on the TAM (Davis 1989), available in the public domain, was adapted with minor contextual word changes to collect the data on attitudes, barriers, PU, PEU and current use of health information technology. The 58-item questionnaire has a four-point Likert scale of agreement with specific positively worded statements related to different types of health information technology. These included: 18 attitude statements, six statements on PU, six statements on PEU, 12 statements on barriers and 16 statements on actual use. The data were collected from November 2019 to March 2020. All statistical analyses were performed using IBM SPSS version 28 (IBM Corporation, Armonk, New York, United States), and significance was set at *p* < 0.05. Likert scales were coded to a binary scale of agree and disagree, and summary scores were calculated for each TAM domain. Regression analysis was conducted to test if PU, PEU, attitudes and barriers predict use of health information technology. The overall tool has adequate internal consistency with a total calculated Cronbach’s alpha of 0.921 (TAM domains: attitudes α = 0.825, PU α = 0.749, PEU α = 0.847, barriers α = 0.837 and use α = 0.911).

### Ethical considerations

The study was approved by the Biomedical Ethics Committee of the University (reference number: BM18/3/2) and permission to conduct the study was also obtained from relevant authorities of Department of Health. Participants were informed about the aims of the study and the procedure of the study. Informed consent was obtained and the participants were informed that participating in the study was voluntary and assured confidentiality and anonymity throughout and beyond the study.

## Results

### Respondent demographics

A total of 93 nurses (58.1% response rate) working in the selected PHC settings at the time of the survey completed questionnaires. Most of the respondents (82, 88.2%) were female, with an average age of 40.9 years (± 10.2), with the youngest being 23 years and the oldest 62 years old. Nearly three quarters of the respondents were professional nurses (67, 72%), 16 were enrolled nurses (17.2%) and only three (3.2%) were enrolled nursing assistants. Just less than half (43, 46.2%) of the respondents had a diploma in nursing, 27 (29%) a degree qualification in nursing and nine (9.7%) an advanced diploma or specialisation. The average number of years of experience was 14.2 years (± 11.5) (median 10 years, ranging from no experience to 36 years).

### Attitudes towards the use of health information technology

To measure attitudes towards the use of health information technology, 18 positively worded attitude statements were rated. Information technology was defined as the use of computers to store, retrieve, transmit or communicate and manipulate patient data or patient information. A total of 17 of the 18 statements had an agreement rate of over 75% of the respondents (Table 1). The three highest rated statements were:

- Computer packages should be used for registration of patient information in health facilities.
- The use of information technology makes it easier to share treatment and care within the health team members.
- Recording the patient’s previous data accurately increases the quality of the intervention, treatment and care planned for the patient.

**TABLE 1:** Agreement with attitude statements about health information technology (*n* = 93).

Attitude statements	*n*	%
Computer packages should be used for registration of patient information in health facilities	89	95.7
The use of information technology makes it easier to share treatment and care within the health team members	89	95.7
Recording the patient’s previous data accurately increases the quality of the intervention, treatment and care planned for the patient	89	95.7
Information technology facilitates patient data sharing between health institutions	88	94.6
The use of computers to record patient information, prevents the loss of patient information	87	93.5
The use of computers assists to ensure that treatment and care are patient-specific	84	90.3
I support the use of Internet in all clinical practice	83	89.2
Computers used in healthcare institutions should be open to facilitate Internet usage	82	88.2
The use of computers in the healthcare contribute to the overall care of patients	81	87.1
Recorded patient data should be used for evaluating the performance of health personnel	81	87.1
The use of the Internet helps me to search for relevant health information to do my clinical work	81	87.1
Technology and information management support the quality and safety of patient care	79	84.9
An electronic health record in a clinical setting assists to plan patient care	78	83.9
Electronic records support the clinical decision-making process	77	82.8
The use of the Internet improves my clinical performance	77	82.8
It is necessary for clinical nurses to use Internet in providing healthcare	72	77.4
Information technology makes it easier for patients to access their own health information	70	75.3
I will not be able to provide effective patient care if I am not using the Internet	36	38.7

Eighty-nine (95.7%) respondents agreed with these statements. The lowest rated statement was ‘I will not be able to provide effective patient care if I am not using the Internet’ (36, 38.7%). There were no significant differences in attitude score for gender or category of nurse.

### Perceived usefulness

To measure PU, six statements on the usefulness of various types of health information technology were rated. Health information systems were defined as systems designed to manage healthcare data: collecting, storing, managing and transmitting patient’s electronic medical records. Overall, all six statements had levels of agreement higher than 70%, with most of the respondents (82, 88.2%) agreeing that ‘using health information systems [*was*] good for workflow and professional development and useful for patient care’ (*n* = 76, 81.7%) (Table 2).

**TABLE 2:** The perceived usefulness of health information technology.

Perceived usefulness statements	*n*	%
Using health information systems is good for workflow and professional development	82	88.2
I would find health information systems useful for my patient care	76	81.7
My interaction with a computer assists me to communicate patient information	73	78.5
Using health information systems increases productivity	72	77.4
Using health information systems enables me to accomplish tasks more quickly	71	76.3
The computer is a tool to assist better nursing care but there are human functions that cannot be performed by computer.	66	71.0

### Perceived ease of use of health information technology

To measure PEU of health information technology, six statements of ease of use and confidence in use were rated. Over three-quarters of the respondents (73, 78.5%) reported that they find it easy to *use computers to analyse patient data and plan patient care*. However, less than two-thirds (54, 58.1%) of the respondents felt ‘confident in their ability to plan patient care by assessing electronic patient record’, and just over half (49, 52.7%) felt ‘confident in their ability to maintain an electronic health record while providing patient care’ (Table 3).

**TABLE 3:** The perceived ease of use of health information technology.

Perceived ease of use statements	Agree = *n*	%
I would find it easy to use a computer for analysing patient data to plan patient care	73	78.5
It is easier for me to use a computer for recording patient data	63	67.7
Learning to operate a computer to process patient data is easy for me	60	64.5
I feel confident in my ability to navigate online health information to provide information about the risk for a patient	56	60.2
I feel confident in my ability to plan patient care by assessing electronic patient records while providing care to reduce the risk to the patient	54	58.1
I feel confident in my ability to do an electronic health record while providing patient care	49	52.7

### Perceived barriers in using health information technology

Twelve statements on barriers were rated, including external barriers, internal barriers of personal skill and beliefs. In terms of the external factors, more than three-quarters (71, 76.3%) of the respondents reported that they did not have Internet access, and 69 (74.2%) reported no access to computers. In rating their own competence, training and skill levels, nearly two-thirds (57, 61.3%) had no training on how to search health information on the Internet for patient care. However, for the rest of the personal skills, less than half of the respondents agreed with these, with 45 (48.4%) lacking experience with computers, 41 (44.1%) having no typing skills, 39 (41.9%) not knowing how to use the Internet and 19 (20.4%) indicating anxiety or fear of computers (Table 4). Some beliefs held by respondents also were rated as barriers to using health information technology, with over half (52, 55.9%) of the respondents believing that searching health information on the Internet can reduce nurse–patient interaction. Over a third (34, 36.6%) of the respondents also agreed that Internet use during working hours decreased productivity (Table 4).

**TABLE 4:** Perceived barriers to using health information technology.

Barriers	Agree = *n*	%
**External barriers**		
I don’t have Internet access	71	76.3
I lack access to a computer	69	74.2
**Personal skills**		
I lack training on how to search health information on the Internet for patent care	57	61.3
Lack of experience in using computer applications	45	48.4
I lack typing skills	41	44.1
I don’t know how to use the Internet	39	41.9
I can’t find what to look for on the Internet	28	30.1
Fear of using computers	19	20.4
**Personal belief barriers**		
I believe that searching health information on the Internet can reduce nurse–patient interaction	52	55.9
A belief that nurses’ use of Internet during working hours decreases productivity	34	36.6
Negative beliefs on the use of Internet technology to assist clinical patient care	30	32.3
It is time consuming to search for health information on the Internet	39	41.9

### Use of health information technology

Use of health information technology was measured using 16 statements, 9 statements on seeking and use of health information and 7 statements on their ability to use health information technology for the administration and management of patient care. Respondents had high ratings for accessing health information, namely to educate patients at risk (78, 83.9%), to improve the patient care process (75, 80.6%) and to facilitate care and identify patient risks (70, 75.3%). There were lower agreement ratings (53, 57%) for ‘knowing how to use healthcare data to reduce risk of disease and for using healthcare data to find solutions for patients at risk of diseases’ (52, 55.9%). This was consistent with the lowest rating for being able to use the computer to conduct online literature searches to support patient care and treatment (49, 52.7%) (Table 5).

**TABLE 5:** Use of health information technology.

Variable	*N*	%
**Seeking and use of health information**		
I know how to find important health information to educate patients at risk of diseases	78	83.9
I use healthcare information to improve patient care processes	75	80.6
I often use patient information to facilitate my clinical care and identify patients at risk	70	75.3
I use healthcare data to understand a health problem or an illness	61	65.6
I often use healthcare data to assess a patient’s condition if they are at risk	59	63.4
I am able to search the Internet to locate and download evidence-based information to educate patients at risk	58	62.4
I know how to use healthcare data to reduce risk of disease	53	57.0
I frequently use healthcare data to find solutions for patients at risk of disease	52	55.9
I am able to use a computer to conduct an online literature search to support patient treatment and care	49	52.7
**Use of heath information technology for administration of patient care**		
I always maintain the privacy of patient health information and patient safety	86	92.5
I am able to use a computer to access patient data, upload, download and e-mail relevant information	57	61.3
I am able to retrieve patient data to assist me with patient care	56	60.2
I am able to use a computer for administrative application of patient information, such as demographics data, billing data	55	59.1
I am able to use a computer to communicate relevant information to patients at risk	50	53.8
I am able to use a computer to capture patient data, for example, vital signs, patient history	46	49.5
I am able to use a computer application to plan care, including discharge planning and information sharing with patients	43	46.2

Overall, apart from using health information to ensure privacy and safety (86, 92.5%), there were low-agreement ratings of respondents’ abilities to use health information systems for patient management and administration. Just over half (57, 61.3%) of the respondents rated that they were ‘able to use a computer to access patient data, upload, download and e-mail relevant information’. The lowest agreement was for being able to ‘use an application to support patient treatment and care’ (46, 49.5%) and being able to ‘use computer applications to plan care, including discharge planning and information sharing with patients’ *(*43, 46.2%).

### Overall technology acceptance and predictions of health information technology use

Overall technology acceptance was measured through the five constructs described here. Significantly moderate positive correlations were found between PU and PEU (*r* = 0.611, *p* < 0.001). The stronger the nurses’ perceptions of usefulness were, the stronger were their perceptions of ease of use. There were no significant differences between the professional groups in all constructs except for the rating of the barriers in technology use, in which the professional nurses scored significantly lower than nonprofessional nurses, respectively (5.1 vs. 7.3, *U* = 2.3, *p* = 0.019), indicating lower levels of barriers. Multiple regression was performed to see how well attitudes, barriers, PU and PEU (independent variables) predict use of health information technology (dependent variable). The full model containing the four predictors was statistically significant, *F* (4, *N* = 50) = 16.1, *p* < 0.001, indicating that the model was able to distinguish between respondents’ use. The model explained between 55.3% of the variance in use of technology, while only one of the independent variables made a unique statistically significant contribution to the model (PEU), explaining 60.9% of the variance in use of technology (*β* = 0.609, *T* = 4.9, *p* < 0.001). A second model with use of data for patient administration and management explained 58.2% of the variance in use of technology, with (again) PEU making a unique statistically significant contribution to the model, explaining 66.7% of the variance in use of technology (*β* = 0.667, *T* = 5.7, *p* < 0.001).

## Discussion

The aim of this study was to describe the current use of health information technology among primary healthcare nurses in the context of technology acceptance of health information technology use. This study measures respondents’ attitudes, perceived use and ease of use of health information technology and barriers experienced in using health information technology (TAM).

### Use of health information technology

In measuring the use or the ability to use health information technology, two categories of use emerged. Firstly, seeking and using data and information for patient education, organising workflow processes and identifying patients at risk. High levels of use were reported for these activities (> 75% agreement). The introduction of healthcare technology can facilitate the healthcare process and can help nurses to offer safe and effective care and reduce the occurrence of missed nursing care (Piscotty, Kalisch & Gracey-Thomas 2015). The findings are also consistent with the study results by Del Carmen Ortega-Navas (2017) that health information technology has brought significant support in health education (Del Carmen Ortega-Navas 2017). However, when measuring the respondents’ self-reported abilities to access and or seek information, just over half of the respondents (50% and 65%, respectively) agreed with these statements. Similarly, in the second category of use, patient administration and management, with activities such as using health information technology to capture patient information, patient administration and patient care, only about half of the respondents agreed with the statement on their ability to use health information technology. The general low ratings of actual ability to use health information technology may be because of the barriers reported in this study, with high levels of lack of access to computers and the Internet and only 40% of the respondents reporting having had training in the use of health information technology. Lower use of health information technology identified were also reported in a study among doctors (40.9%), students (25%) and health staff (38.7%) (Sadoughi & Erfannia 2017).

The only exception in the relatively low rating of their ability to use health information technology was maintaining patient information and patients’ safety, with nearly all respondents reporting that they always keep privacy of patient health information and patient safety. Petersen (2018) observed that patient information privacy and safety is a regulatory requirement achieved by all healthcare providers and that the privacy of patient information management needs particular consideration during health information sharing (Petersen 2018).

### Attitudes towards use of health information technology

As nurses are the key players in integrating health information technology in the provision of primary healthcare, it was encouraging to note that the respondents presented a positive attitude towards the use of health information technology, with more than 75% of the respondents agreeing with the statements. The exception was that in this study (and like a study by Kuek and Hakkennes [2020]), about 20% of the respondents reported anxiety about the use of information technology. Although this study did not explore this, Top and Yilmaz (2015) suggested that self-efficacy, affective feelings, computer literacy and negative beliefs could contribute to fear (Top & Yılmaz 2015).

The overall finding of positive attitudes is similar to other nursing studies, which found that healthcare staff reported positive attitudes towards information systems (Gürdaş Topkaya & Kaya 2015; Kuek & Hakkennes 2020). Nearly all of the respondents (95.7%) were positive about the use of computer packages for registration of patient information, which was also consistent with other similar study findings where nurses reported that the use of health information technology improves the quality of care (Gilmour et al. 2016). This finding, however, was in contrast with the reported low level of ability of the respondents to use health information technologies for patient administration and management.

### Perceived ease of use and perceived usefulness of health information technology

The study demonstrated positive perceptions of the usefulness of health information technologies, with high levels of agreement for the use of health information technologies for patient administration (88.2%), analysis and planning patient care (81.7%) and communication with patients (78.5%). This is consistent with a study reporting that nurses perceived the use of health information systems for patients’ data collection and documentation, allowing nurses to spend more time on patient care while improving accessibility and efficient documentation (Huang 2021). This finding, however, was also in contrast (again) with the reported low level of ability of the respondents to use health information technologies for patient administration and management.

Perceived ease of use were also positive, although less so than their perceptions of usefulness, with only ‘ease of use of computers for analysing patient data to plan’ having more than 70% agreement. The lower perceptions of ease of use are more consistent with the lower reported levels of ability of the respondents to use health information technologies for patient administration and management. Ease of use and usefulness were significantly moderately correlated and were the only significant constructs that explained about 60% of the use of health information technology. This is similar to other studies, which also found strong correlations and ease of use directly affecting PU (Tubaishat 2018).

### Barriers to using health information technology

As indicated, the main reported barrier to health information technology use was access to computers and the Internet and a lack of training. Over three-quarters of the respondents (76.3%) did not have access to Internet, 74.2% reported a lack of access to computers, 61.3% lacked training on how to search health information on the Internet and 42% of nurses did not know how to use the Internet. Increased access to computers and Internet by nurses is essential as it enables them to retrieve information related to clinical practice (Ahmad et al. 2018). A similar study conducted in primary healthcare in other low-income countries has shown that the adoption of health information technology is hampered by insufficient IT infrastructure, human resources, organisational support and processing factors (Afrizal et al. 2019). Similarly, in a review of m-health technologies in primary healthcare, the main challenges health workers experienced were poor network connections, access to electricity and the cost (Odendaal et al. 2020). The findings of this study are also supported by Steele Gray et al. (2018), who argue that the barriers to the use of technology in primary care are linked to information access barriers, limited functionality of available technology and organisational and provider inertia (Steele Gray et al. 2018).

### Limitations

The study has several limitations. Firstly, the use of a self-reported questionnaire often has self-reflection bias. The second limitation of the study was the small sample size and a nonrandom sampling technique that was used to select the respondents. Although useful for the actual setting, this could constitute a threat to the external validity of the results, and the findings may not be generalisable to a similar population and context.

## Conclusion

Health workers require access to health information technology, training, technical support, user-friendly devices and systems that are integrated into existing electronic health systems (Odendaal et al. 2020). The respondents reported positive attitudes towards the use of health information technology and positive perceptions of usefulness and ease of use of health information technology. However, barriers of access, individual skill and training remain high in this setting. Actual health information technology use was varied and was best predicted by perceptions of PEU. This study adds to the body of knowledge of technology acceptance in nursing and specifically begins to provide a picture for health service planners of the readiness for health information technology use and acceptance in primary healthcare nurses in this setting and the continued lack of access to computers and Internet in primary healthcare settings.

### Recommendations

Providing proper and adequate technology infrastructure (access to Internet and computers) and frequently updated training for nurses are recommended. Demonstrating the usefulness of health information technology is vital for clinical practising nurses in primary healthcare and to ensure patient benefits.

This study should be expanded to include a cross-sectional study of all primary health services to identify the current access and training needs for the integration of health information technologies. This study can also be complemented with qualitative interviews to explore issues of fear and anxiety with regard to technology use.
